# Decrease in shunt volume in patients with cryptogenic stroke and patent foramen ovale

**DOI:** 10.1186/1471-2377-10-123

**Published:** 2010-12-29

**Authors:** Christian Tanislav, Manfred Kaps, Marek Jauss, Erwin Stolz, Wolfgang Pabst, Max Nedelmann, Mathias Grebe, Frank Reichenberger, Jens Allendoerfer

**Affiliations:** 1 Department of Neurology, Justus Liebig University, Giessen, Germany; 2 Department of Neurology, Oekumenisches Hainich Klinikum Muehlhausen, Germany; 3 Institute for Biomedicine and Epidemiology, Justus Liebig University, Giessen, Germany; 4 Department of Cardiology, Justus Liebig University, Giessen, Germany; 5 Department of Pulmonology, Justus Liebig University, Giessen, Germany

## Abstract

**Background:**

In patients with patent foramen ovale (PFO) there is evidence supporting the hypothesis of a change in right-to-left shunt (RLS) over time. Proven, this could have implications for the care of patients with PFO and a history of stroke. The following study addressed this hypothesis in a cohort of patients with stroke and PFO.

**Methods:**

The RLS volume assessed during hospitalisation for stroke (index event/T0) was compared with the RLS volume on follow-up (T1) (median time between T0 and T1 was 10 months). In 102 patients with a history of stroke and PFO the RLS volume was re-assessed on follow-up using contrast-enhanced transcranial Doppler/duplex (ce-TCD) ultrasound. A change in RLS volume was defined as a difference of ≥20 microembolic signals (MES) or no evidence of RLS during ce-TCD ultrasound on follow-up.

**Results:**

There was evidence of a marked reduction in RLS volume in 31/102 patients; in 14/31 patients a PFO was no longer detectable. An index event classified as cryptogenic stroke (P < 0.001; OD = 39.2, 95% confidence interval 6.0 to 258.2) and the time interval to the follow-up visit (P = 0.03) were independently associated with a change in RLS volume over time.

**Conclusions:**

RLS volume across a PFO decreases over time, especially in patients with cryptogenic stroke. These may determine the development of new strategies for the management in the secondary stroke prevention.

## Background

Several studies identified an increased frequency of patent foramen ovale (PFO) in patients with stroke of undetermined aetiology, also referred to as cryptogenic stroke (CS) [1,2]. Nevertheless the clinical relevance of PFO in cerebral ischemic disease is still a topic of debate [3,4]. In patients with PFO, the proposed mechanism for stroke is paradoxical embolism (PDE) [5,6]. PDE diagnosis can be regarded as definitive if observed during transesophageal echocardiography (TEE), as well if a thrombus within the PFO is found on autopsy [7-9]. It is therefore difficult to establish a cause-and-effect relationship between PFO and PDE [10,11], resulting in a lack of clear guidelines for appropriate management to prevent recurrent stroke [12,13].

If PDE is the suspected cause of stroke, and a physiologic trajectory of PFO throughout life-time is considered, secondary prevention measures should target further PDE events. However, there have been anecdotal cases reporting a reduction in RLS volume, for instance during treatment for massive pulmonary embolism (PE) [14,15]. Whether shunt volume in patients with stroke and PFO may change over time has not yet been proven.

The purpose of the present exploratory study was to investigate a potential RLS change over time in a cohort of patients with a history of stroke and PFO. Proven, it may be regarded a pivotal finding, with an impact on the development of new strategies for management of these patients including an individualized delivery of care regarding secondary prevention.

## Methods

### Patients

Potential patients for inclusion in the study were identified using case notes (from January 2002 to June 2007). Eligible for study entry were patients who fulfilled the following criteria: stroke or transient ischemic attack (TIA), RLS and PFO proven by TEE and contrast-enhanced transcranial Doppler/duplex ultrasound (ce-TCD), past index event at least one month prior to study entry.

Reviewing the case notes 169 consecutive patients with stroke and PFO were identified. As ce-TCD detects any type of RLS, irrespective of its location in the vascular system, we decided to include only patients who had undergone both TEE and ce-TCD. 130 (77%) of the original 169 patients were identified as eligible for study inclusion.

The study protocol was reviewed and approved by the local ethical committee. Patients gave informed consent prior to study participation.

### Study design

In this mono-centre observational study eligible patients were contacted by telephone and invited to participate in the study. All patients gave consent to the study protocol. To re-assess the shunt volume across the PFO, a ce-TCD was performed. The examiner carrying out the ce-TCD did not have access to clinical data and initial measurements. On follow-up, further information was recorded for each patient: number, date and type of cerebrovascular events after the index event (all events were validated by obtaining the relevant case notes), treatment received during follow-up period, disability status as assessed by modified Rankin scale (mRs), time interval between the index event and follow-up visit (T0-T1) in months. Baseline demographic and clinical information were obtained from case notes recorded at the index event.

### Setting

A change in shunt volume was defined as a difference of ≥20 microembolic signals (MES) or no evidence of RLS on follow-up examination. Serial ceTCDs performed on two subsequent days in 18 individuals with PFO, shunts in countable range revealed an intraindividual difference of ≤12 MES (unpublished data). Based on this observation the cut-off ≥20 MES was established. In 76% of patients a RLS on T0 was not evident without Valsalva. For proving a change in RLS, results obtained under Valsalva strain were therefore considered for the analysis.

## Instrumentation

### ce-TCD

A 2 MHz probe (Philips HP SONOS 5500, Philips Healthcare, Hamburg, Germany) was used to carry out ce-TCD. The contrast agent, based on a D-galactose microparticle solution (Echovist™, Bayer Vital, Berlin, Germany), was prepared according to the manufacturer's instructions, and injected into a large cubital vein of the left arm. The contrast agent was administered as two separate boluses of 5 ml each, one while normal respiration and the other while a 10 seconds Valsalva strain. The Valsalva manoeuvre was practiced with the patient before the procedure. In line with the recommendations of the international consensus meeting, the RLS was classified according to the number of MES that appeared in the cerebral circulation (insonation of the proximal part of the left middle cerebral artery) as minimum (1-10 MES), moderate (11-30 MES) or massive (> 30 MES) or defined as a curtain pattern if an individual MES count was not possible [16]. Assessed in our department, repeated ce-TCD examinations revealed a concordance rate as follows: minimum shunts of 100%, medium shunts of 95% and massive shunts of 100% respectively (unpublished data). Examinations are routinely recorded on a CD-ROM. All examinations (T0 and T1) were evaluated offline by two experts in a standardized protocol, blinded from any clinical data and the individual chronological order. In case evaluations were graded differently (different MES count), a consensus read was undertaken.

### TEE

TEE was performed by experienced echocardiographers from the Department of Cardiology using a 4-7 MHz multi-plane probe (HP Sonos 5500, Philips Healthcare, Hamburg, Germany). The same examiner carried out 95% of the TEEs.

To detect an intracardiac shunt, 10 ml of the contrast agent (Echovist™, Bayer Vital, Berlin, Germany) was administered by bolus injection into a large antecubital vein. RLS was evident if the transit of microbubbles from the right to the left atrium occurred spontaneously or during a subsequent Valsalva manoeuvre. PFO was diagnosed when at least three microbubbles were detected in the left atrium within three heart beats after appearance in the right atrium. The PFO classifications used were minimum (3-10 microbubbles), moderate (11-30 microbubbles) and massive (> 30 microbubbles). An atrial septal aneurysm (ASA) was diagnosed when the atrial septum extended at least 11 mm into the left or the right atrium, or both. An excursion of minimum 5 mm of the septum primum into either the left or right atrium with respect to a perpendicular line to the fossa ovalis plane was considered as hypermobile atrial septum.

### Statistical evaluation

If shunt volume changed between T0 and T1, an analysis was carried out to determine whether the change was associated with: age, sex, index event classified as cryptogenic stroke, use of oral anticoagulants, diagnosis of deep vein thrombosis (DVT) on index event, diagnosis of either ASA or hypermobile atrial septum and the time delay from index event to follow-up assessment as calculated in months (T0-T1).

Non-parametric data were compared using the Mann-Whitney 2-tailed U-test. Comparisons of dichotomized data between the index event and follow-up were analyzed using Fisher's exact test. Potential factors associated with a change in shunt volume were identified by logistic regression analysis. For the statistical analysis the SPSS Software (Statistical Package for Social Sciences) release 15.0 (SPSS Inc., Chicago, IL, USA) was used.

## Results

### Sample

All patients eligible for the study were contacted by phone (n = 130). Nine patients (6.9%) were no longer eligible as they had already undergone a PFO closure. Six patients (4.6%) could not be contacted, and 11 (8.5%) declined to participate in the study. Two patients (1.5%) were excluded from a study participation for logistical reasons (the catheter used for the injection of contrast agent was unsuitable). In total, 102 patients (78.5%) were included in the study.

The median and mean age of the study population analyzed was 55.4 and 53.3 years (range, 19.7-80.2 years). Sixty-two patients were male (60.8%). According to classification criteria published previously [17], 39 patients (38.2%) were classified as having cryptogenic stroke (Table 1); they were predominantly treated with oral anticoagulation (28/39, 71.8%). In 5 patients (1.8%) a DVT was evident at the index event.

**Table 1:** Distribution of aetiologies according to the TOAST classification

TOAST classification (n = 102)	Number of patients (%)
Small artery or lacunar stroke	23 (22)
Large-artery atherosclerosis	16 (16)
Cardioembolism	16 (16)*
Uncommon, known causes of stroke	8 (8)^†^
Undetermined (cryptogenic) stroke	39 (38)

*patients additionally diagnosed of atrial fibrilation

^†^includes causes such as artery dissection, vasculitis and anti-phospholipid antibodies.

### Initial PFO and RLS assessment

At the index event, in TEE 74 patients (72.5%) had a massive RLS; in ce-TCD 72 patients (70.6%) had a large RLS. An ASA was identified in 23 patients (22.5%), and a hypermobile atrial septum was identified in 26 patients (25.5%). At least one septal abnormality was identified in 35 patients.

Overall, there was a 95% agreement between the size of the RLS identified using ce-TCD and TEE (97/102 patients). Among patients with massive RLS, the concordance rate between ce-TCD and TEE was 97.2% (72/74 patients) (Table 2).

**Table 2:** Shunt size concordance TEE vs. ce-TCD assessed at index event (n = 102)

Shunt size	TEE at index event	ce-TCD at index event	Concordance (%)
Large	74	72	72/74 (97.2)
Medium	16	19	16/19 (84.2)
Minimum	12	9	12/9 (75)
Total			97/102 (95.1)

### Follow-up RLS assessment

The follow-up assessment was performed at a median of 10 months after the index event. The mean follow-up time was 12.5 months. The follow-up period totalled 1277 patient-months of observation. During the follow-up period, five patients (5%) had a recurrence event (2 transient ischemic attacks and 3 strokes); 40 patients (39.2%) received oral anticoagulants. The median disability score as assessed using mRs was 1 (range: 0-3).

A small increase (difference ≤8 MES) in shunt volume was detected in 12 patients (median 5 MES, range 3-8 MES). These patients were recorded as having an unchanged shunt volume. In total, there was no evidence of a change in shunt volume between the index event and follow-up in 71 patients (70%).

Table 3 shows the RLS change in 31 patients who revealed a reduction. Seventeen of these patients (54.8%) initially presented with a curtain. In 14 patients (45.2%) the RLS was no longer detectable on follow up. Eleven patients (35.5%) initially assessed as having a curtain pattern revealed 3-14 MES on follow-up. A reduction from curtain pattern to no evidence for RLS was found in 6 patients (19.4%). In these patients the ce-TCDs was repeated three times within the subsequent four months. In all examinations no RLS could be detected. None of the succeeding examinations revealed a RLS. As in 3 of these patients (9.7%) a PFO-closure was considered as a therapeutic option, they even underwent a TEE, revealing regular findings and confirming the preceding ce-TCDs results.

**Table 3:** PFO shunt volumes assessed by ce-TCD at index event and follow-up visit in 31 patients who showed a reduction (n = 102)

Patient number	ce-TCD at index event (MES)	ce-TCD at follow-up (MES)	T0-T1 (months)
1	16	0	24
2	Curtain	0	26
3	Curtain	0	5
4	20	0	8
5	Curtain	0	6
6	Curtain	0	26
7	30	0	5
8	40	0	26
9	Curtain	0	24
10	20	0	3
11	25	0	36
12	15	0	12
13	Curtain	0	5
14	13	0	3
15	35	3	12
16	Curtain	3	20
17	25	3	2
18	25	3	12
19	Curtain	3	3
20	Curtain	5	40
21	Curtain	5	60
22	Curtain	5	12
23	40	6	24
24	36	6	60
25	Curtain	7	1
26	40	7	8
27	Curtain	8	1
28	Curtain	9	12
29	Curtain	9	36
30	Curtain	12	2
31	Curtain	14	48

'Curtain' refers to a shower of microembolic signals (MES): too many to be counted individually

### Inferential statistics

For the entire study 204 ce-TCD examinations were re-evaluated offline. In examinations with curtain pattern (n = 62, 30.4%) and those with no evidence for RLS (n = 14, 6.9%) an interrater agreement of 100% between the 2 experts was achieved. In the remaining 128 (62.7%) ce-TCD examinations with shunts in a countable range a consensus read was necessary in 5 cases. For ce-TCDs in a countable range, the interrater reliability for the MES count was calculated, resulting in a remarkably high value (kappa = 0.95, CI = 0.93-0.97; *P *< 0.001).

Univariate analysis showed an association between change in shunt volume and age, sex, presence of ASA, index stroke classified as cryptogenic and use of oral anticoagulants and. Factors initially associated in the univariate analysis were entered into a logistic regression. Finally the time delay T0-T1 as calculated in months from index event (*P=*0.03) and a cryptogenic stroke (*P *< 0.001; OD = 39.2, 95% confidence interval 6.0 to 258.2) were identified as independent factors associated with a change in RLS (Table 4).

**Table 4:** Relationship between selected parameters and change in right-to-left shunt (RLS) in patients with patent foramen ovale and a history of stroke

	Total patient cohort n = 102 (%)	RLS volume change n = 31 (%)	No RLS volume change n = 71 (%)	P*	***P***^†^
Age (years) median/(range) mean/(SD)	55.4/(19.7-80.2) 53.3/(15.77)	44.3/(19.7-74.4) 37.7/(14.85)	58.9/(21.4-80.2) 57.1/(14.73)	< 0.001^‡^	0.881
Sex				0.04^§^	0.685
Female	40 (39.25)	17 (54.8)	23 (32.4)		
Male	62 (60.8)	14 (45.2)	48 (67.6)		
Cryptogenic stroke	39 (38.2)	27 (87.1)	12 (16.9)	< 0.001^§^	< 0.001
Use of anticoagulants	40 (39.2)	21 (67.7)	19 (26.8)	< 0.001^§^	0.969
Atrial septum aneurysm (ASA)	23 (22.5)	11 (35.5)	12 (16.9)	0.069^§^	0.165
Hypermobile atrial septum	26 (25.5)	7 (22.6)	19 (26.8)	0.806^§^	
Deep vein thrombosis (DVT)	5 (4.9)	1 (3.2)	4 (5.6)	0.999^§^	
T0-T1 (months) median/(range) mean/(SD)	10/(5-60) 12.5/(12.1)	12/(7-60) 15.6/(12.02)	9/(5-32) 10.17/(8.1)	0.081^‡^	0.037

*P value calculated in univariate analysis

^†^*P *value calculated in the logistic regression analysis

^§^*P *value based on Fisher's exact test

^‡^*P *value based on two-tailed Mann-Whitney *U*-test

In a subgroup analysis in patients with cryptogenic stroke versus patients with determined aetiology, we found a significant association of RLS reduction with an index event of cryptogenic stroke (Table 5); 27 of 39 patients (69.2%) with cryptogenic stroke had a reduction of RLS or functional closure of the PFO, compared to 4 of 63 patients (6.3%) with a stroke of known aetiology (*P *< 0.001; OD = 31.2, 95% confidence interval 5.7 to 170.6). In this context, it is important to note that there was no difference between the two groups as for the time delay (T0-T1) on follow-up. Further independent factors associated with cryptogenic stroke were: use of oral anticoagulants (*P *< 0.001; OD = 10.1, 95% confidence interval 2.2 to 46.7) and young age (*P *= 0.003).

**Table 5:** Factors associated with cryptogenic stroke; subgroup comparison of patients with cryptogenic stroke versus patients with other aetiology

	Cryptogenic stroke n = 39 (%)	Other aetiologies n = 63 (%)	P*	***P***^**†**^
Age median/(range) mean/(SD)	40/(19.7-65.2) 41.9/(14.5)	63/(34-80.2) 60.4 (11.9)	< 0.001^‡^	< 0.001
Sex			< 0.001^§^	0.102
Female	24 (61.5)	16 (25.4)		
Male	15 (38.5)	47 (74.6)		
RLS shunt size reduction	27 (69.2)	4 (6.3)	< 0.001^§^	< 0.001
Use of anticoagulants	28 (71.8)	12 (19)	< 0.001^§^	0.003
Atrial septum aneurysm (ASA)	11(28.2)	12 (19)	0.33^§^	
Hypermobile atrial septum (HAS)	10 (25.6)	16 (25.4)	< 0.999^§^	
ASA or HAS	16 (41)	19 (30.2)	0.29^§^	
Deep vein thrombosis (DVT)	3 (7.7)	2 (3.2)	0.37^§^	
T0-T1 median/(range) mean/(SD)	12/(6-60) 14.9/(14.4)	10/(5-45) 11.1(10.2)	0.3^‡^	

*P value calculated in univariate analysis

^†^*P *value calculated in the logistic regression analysis

^§^*P *value based on Fisher's exact test

^‡^*P *value based on two-tailed Mann-Whitney *U*-test

There was no difference in stroke recurrence between patients with RLS change on follow-up versus without a RLS change (1/31, 3.2% vs. 4/71, 5.6%; *P *> 0.99). Patients treated with oral anticoagulation were free of recurrences during the follow-up period (0/40, 0% vs. 5/62, 8.1%; *P=*0.15).

## Discussion

Our primary finding is a reduction in RLS volume over time in 30% of stroke patients with proven PFO. A reduction of RLS volume or even a functional closure of the PFO was almost exclusively found in patients with cryptogenic stroke.

Changes in RLS across a PFO have also been proven by other investigators, while they focused other aspects. Anzola *et al*. investigated predictors for recurrent stroke in patients with PFO and RLS [18]. When starting the observation period the shunt volume was re-evaluated using ce-TCD. In 8% of the patients a RLS was no longer detected on re-examination [18], a result, which is in line with our findings (13%). The difference (8% vs.13%) may be explained by different baseline characteristics of the selected populations.

Further evidence for a change of shunt volume over time can be derived from studies comparing ce-TCD and TEE for the detection of a PFO. The highest concordance rate in shunt volume was achieved when the two procedures were carried out simultaneously [19,20]. Therefore, even when a different subsequent examination quantifies a different RLS size, this change in shunt volume could be considered a true and accurate finding. Although we could not directly prove shunt constancy during the period of hospitalization, the high concordance rate (95%) between TEE and ce-TCD during hospitalization is an argument for such constancy in the immediate post stroke phase.

In our study a shunt reduction across a PFO was found in a high percentage of patients. It was predominantly evident in patients with stroke of undetermined aetiology. This might suggest an increased likelihood for an involvement of "dynamic" PFOs in the mechanism of PDE. For instance a moderate pressure increase in the right atrium could facilitate an "opening" of a functionally closed PFO. A functional PFO opening/closure was already observed during the course and treatment of an acute massive PE [21,22]. In this context silent PE could be suspected to cause a moderate increase in right atrial pressure as it commonly occurs in patients with venous embolic diseases [23,24]. While in patients with DVT silent PE occurs in 30-60% of the cases [25-29], the frequency of silent PE in patients with PFO-related stroke is unknown. In our study a DVT diagnosed while hospitalization was not associated with shunt dynamic and a screening for silent PE was not systematically performed. Therefore, a further clarification of the potential relationship between silent PE, PDE and shunt dynamic is of high priority. Apart from direct therapeutic consequences, it would help to provide a better understanding of the mechanism of PDE and PFO, respectively.

Several reports have emphasized that the volume of RLS is a crucial risk factor for paradoxical brain embolism in stroke patients. A large shunt volume in patients with PFO and a history of stroke may be associated with stroke recurrence [18,30,31]. Even though our results do not prove an association with lower recurrence rates in case of shunt reduction, the study design does not allow a robust analysis regarding the risk of recurrence.

As therapeutic recommendations in patients with stroke and PFO, so far are based on limited evidence [12,32,33], our results add relevant information on further development of new treatment strategies for secondary prevention. One single management approach, including oral anticoagulants, anti-platelet agents, and percutaneous or surgical closure has not yet been proven to be superior to the other [12,32-34]. Knowledge of a potential reduction in shunt volume over time could be an important aspect when selecting a management strategy for an individual patient. In particular, the spontaneous and functional closure of PFO would be of major clinical importance, with a potential impact on an individualised decision for or against an invasive intervention.

Due to its design, the present study is limited regarding a precise determination of the time interval over which shunt size dynamic is likely to occur. This might be important when scheduling patients for PFO closure. Although we demonstrated a significant association between the time delay (median 12 versus 9 month) and regression of the PFO shunt size, a definitive conclusion is not possible.

The shunt evaluation on T0 represents one of the main limitations of the present study. Even according to the study protocol the ce-TCDs were re-evaluated offline, the examinations at T0 were performed under clinical routine conditions. Given that in our department ce-TCDs are performed strictly standardized, this allows us to consider the data obtained for T0 as robust. A high reliability of detected shunts on T0 is also supported by the remarkable shunt size concordance compared to RLS assessment on TEE.

A further limitation of the present study might be related to a methodologically determined variance of a testing procedure; our results could be interpreted as differences in testing between T0 and T1 versus an actual change in shunt size. In a small case series including patients suffering of migraine (n = 8) who were also diagnosed of PFO, we observed no relevant changes in RLS in consecutive examinations (2 ceTCDs within 6 months) (unpublished data). Even though this is not an appropriate control group for comparison in our study, however these findings support the assumption that the observed shunt reduction is caused by a physiological process. Furthermore, in 6 patients with reduction from an initial curtain pattern to subsequently no RLS at T1, following consecutive ce-TDCs (3× in 4 month) confirmed the result detected at T1. Likewise, a subsequent TEE performed in three of these patients confirmed the functional closure; a thrombus within the PFO, also potentially able to lead to PFO closure, was not observed. Albeit based on few selected cases, these findings may definitively strengthen our study results as accurate findings which are not determined by methodological differences of testing procedures.

Although our study includes a rather small number of patients, the results are distinct in indicating an increased likelihood for shunt reduction, especially in patients with PFO and CS. Even when considering the lower range of the large confidence interval, a six-folded probability to experience a shunt reduction in case of PFO and CS appears an intriguing result.

Considering that a shunt reduction in these patients is of high clinical relevance, further research should focus on the identification of factors involved in the process of shunt dynamic. Especially circumstances which potentially maintain a functionally "closed" PFO as well as factors involved in "opening" of a PFO are of particular interest. In our study factors such as age and use of anticoagulants were associated with stroke type rather than change in shunt volume.

## Conclusions

The present study demonstrates a decrease in PFO shunt volume over time, with a preference to patients with cryptogenic stroke. As a consequence, prior to PFO closure, a re-examination of patients with respect to shunt persistence appears necessary. Even if our results are not yet sufficient to settle a definite therapeutic strategy for an individual patient, they can be considered to be a starting point. As it might have relevant implications in the development of new treatment strategies, further studies are promptly required for verifying our results. In this context a rigorous time schedule for the follow-up assessment and a standardized setting for RLS evaluation are major methodological issues which need to be taken into consideration when planning further investigations.

## Competing interests

The authors declare that they have no competing interests

## Authors' contributions

CT, JA and MG carried out the data collection and drafted the manuscript. CT and JA participated in conception and design. CT and JA conducted the study and provide the data collection. WP and CT performed the statistical analyses. All authors were involved in the analysis and interpretation of the results. MK, MN, MJ, ES and FR revised the manuscript critically for important intellectual content and helped to draft the manuscript. All authors read and approved the final manuscript.

## Abbreviations list

PFO: patent foramen ovale; RLS: right to left shunt; T0: time at index event; T1: time at follow-up assessment; T0-T1: time interval between the index event and follow-up assessment; ce-TCD: contrast-enhanced transcranial Doppler/duplex ultrasound; PDE: paradoxical embolism; TEE: transoesophageal echocardiography; ASA: atrial septum aneurysm; PE: pulmonary embolism; DVT: deep vein thrombosis; MES: microembolic signals; TIA: transient ischemic attack; mRs: modified Rankin scale

## Pre-publication history

The pre-publication history for this paper can be accessed here:

http://www.biomedcentral.com/1471-2377/10/123/prepub
